# A Novel Mutation in *NLRP7* Related to Recurrent Hydatidiform Mole and Reproductive Failure 

**DOI:** 10.22074/ijfs.2019.5657

**Published:** 2019-04-27

**Authors:** Jafar Fallahi, Vahid Razban, Mozhdeh Momtahan, Mojgan Akbarzadeh-Jahromi, Bahia Namavar-Jahromi, Zahra Anvar, Majid Fardaei

**Affiliations:** 1 Department of Molecular Medicine, School of Advanced Medical Sciences and Technologies, Shiraz University of Medical Sciences, Shiraz, Iran; 2 Department of Obstetrics and Gynecology, School of Medicine, Shiraz University of Medical Sciences, Shiraz, Iran; 3 Fetal and Maternal Research Center, Pathology Department, School of Medicine, Shiraz University of Medical Science, Shiraz, Iran; 4 Infertility Research Centre, Shiraz University of Medical Sciences, Shiraz, Iran; 5 Department of Genetics, School of Medicine, Shiraz University of Medical Sciences, Shiraz, Iran

**Keywords:** Hydatidiform Mole, KHDC3L, NLRP7

## Abstract

**Background:**

Hydatidiform mole (HM) is an abnormal human pregnancy with excessive trophoblastic proliferation and abnormal embryonic development, dividing into two complete HM (CHM) and partial HM (PHM) groups. One subcategory of the CHMs is recurrent and familial, which is known as biparental HM (BiHMs) or recurrent HM (RHM). *NLRP7, KHDC3L* and *PADI6* are maternal-effect genes involved in RHMs. *NLRP7* is a major gene responsible for RHMs. This study was performed on patients with molar pregnancies and miscarriage. The aim of this study was to genetic screen for mutations in *NLRP7* and *KHDC3L* genes in an affected woman with previous history of 5RHM and the sibling with history of miscarriage.

**Materials and Methods:**

In this experimental study, DNA was extracted from blood samples. KHDC3L and NLRP7 were polymerase chain reaction (PCR) amplified. The PCR products were purified and Sanger sequenced.

**Results:**

In this study, there is no mutation in *KHDC3L* gene but a novel mutation was identified in the NACHT do- main of *NLRP7* gene. Patient with five recurrent moles had this mutation in the homozygous state while her sister with one miscarriage and one normal child showed this mutation in the heterozygous state.

**Conclusion:**

In this study, we identified a new mutation in *NLRP7* gene of a patient with recurrent HM. Following egg donation, this patient has a normal boy. The sister of this patient with heterozygous mutation has a spontaneous abortion and one normal child that confirm the impact of a defective allele of *NLRP7* on reproductive wastage in a recent finding.

## Introduction

Hydatidiform mole (HM) is an abnormal human conception with a defect in fetal development and growth (1). HM is divided into two categories, complete HM (CHM) and partial HM (PHM). CHMs are commonly androgenetic diploid conceptions (2) and PHMs are mostly dispermic triploid conceptions (3). Both CHM and PHM have an extra set of the paternal genome, therefore, paternal genes are more expressed and consequently show excessive trophoblastic proliferation (4). In most of the cases, HM is sporadic, however, in a subgroup of CHM, it is recurrent and familial condition which is known as biparental HM (BiHMs) or recurrent HM (RHM) (OMIM 231090). Occurrence of at least two moles in the same woman is referred to recurrent type and this form is inherited in an autosomal recessive fashion. Frequency of RHMs in the Middle and Far East is reported about 2.5% up to 9.4% of all HMs, which is twice or more compared to Western countries (5-9).

So far, three maternal-effect genes, *NLRP7, KHDC3L* and recently *PADI6*, have been identified to be responsible for RHMs (10-12). It is suggested that these three genes function in setting genomic imprinting process (13). *NLRP7* mutations have been reported in 48-80% of RHMs cases (14-19), while mutations in *KHDC3L* was only reported in 10-14% of these patients with no NLRP7 mutations (10, 20, 21). Homozygote or compound heterozygote mutations of these three genes have been observed in most of the affected women (22). There is still a few fractions of RHM patients with the unidentified responsible gene. *NLRP7* is the principal gene responsible for RHMs, identified by Murdoch and colleagues in 2006. *NLRP7* as the candidate of maternal-effect gene is responsible for RHMs and reproductive disorders such as spontaneous abortions and stillbirths (11).

**Fig 1:**
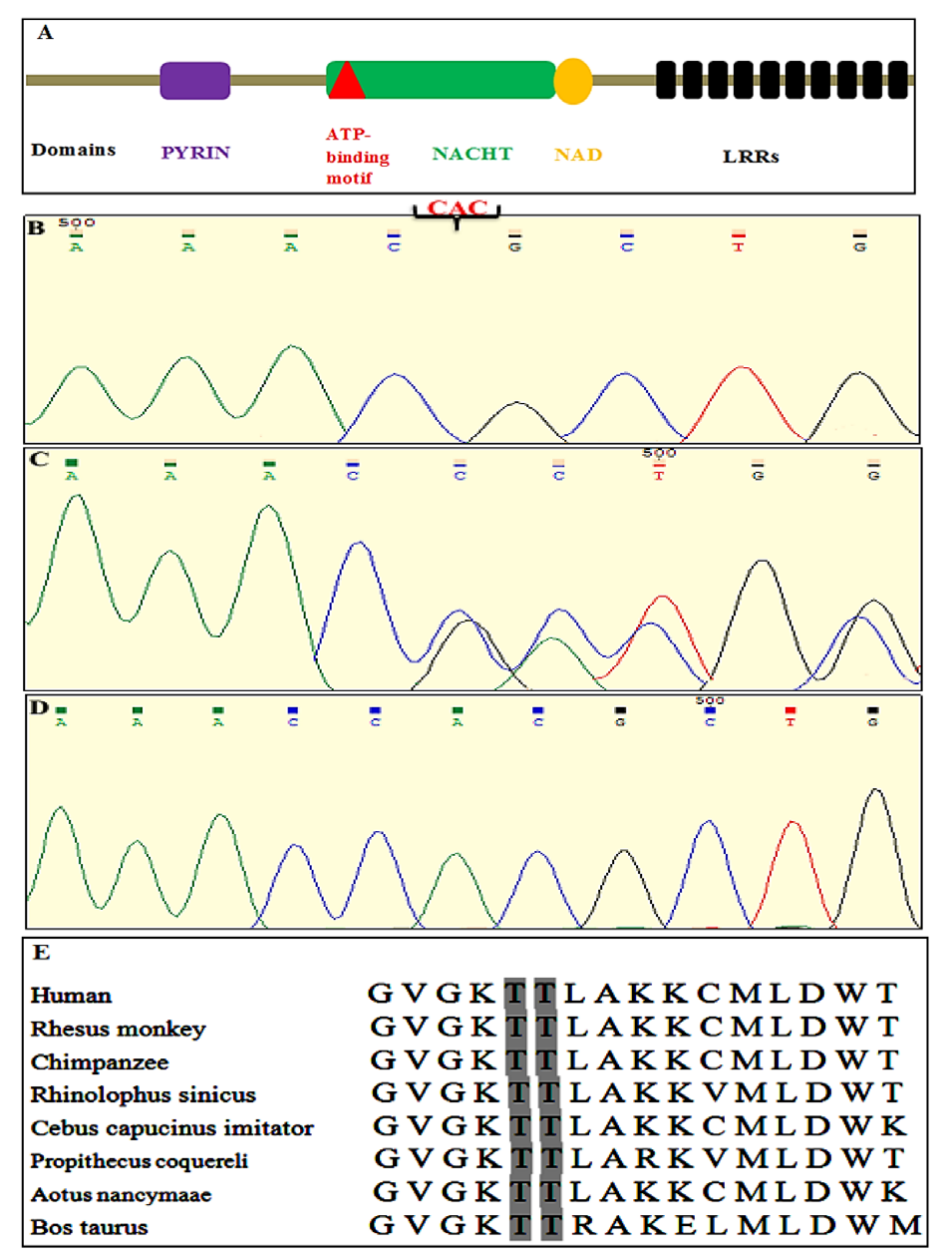
Deletion of the highly conserved Threonine amino acid from NACHT domain. **A.** NLRP7 protein domains including PYRIN, NACHT, NAD, leucine-rich repeats and ATP binding motif in the NACHT domain is depicted, **B.** Sequence chromatogram show deletion of CAC nucleotides in homozygote state in the patient’s *NLRP7* gene, **C.** Heterozygote deletion of CAC nucleotides in her sister, **D.** Normal allele in the wild type individual, and *E.* Threonine 185 residue is highly conserved during evolution.

*NLRP7*, which encodes a protein with 1037 amino acids, is a member of the CATERPILLER protein family with four conserved and functional pyrine, 9-10 leucine-rich repeats, NACHT-associated domain (NAD) and a NACHT domain (Fig .1A) (23, 24). About 48% of intronic sequences of *NLRP7* gene contain Alu repetitive elements. It is believed that Alu repeats act as a hot spot for INDEL mutations (20). To date, 60 pathogenic point and *INDEL* mutations have been reported in NLRP7 (20, 25). In this study, a new mutation was identified in *NLRP7* gene in a patient with recurrent HM. This patient has a normal boy using egg donation. Also, the sister of this patient with heterozygous mutation has a spontaneous abortion and one normal child.

## Materials and Methods

In this experimental study, two sisters with molar pregnancies and miscarriage referred to the Infertility Center in Shiraz University of Medical Sciences, Shiraz, Iran. In the patient, as proband, five moles were reported without any normal child. Patient’s sister represented one normal child and one miscarriage. Proband was diagnosed as BiHMs because she has more than two moles and genetic studies were performed on *NLRP7* and *KHDC3L* genes. Genomic DNA was isolated from whole blood cells using DNA Kit (Cinnaclon, Iran). Three exons and intron boundaries of *KHDC3L* and 11 exons and intron boundaries of NLRP7 were polymerase chain reaction (PCR) amplified using our previously designed primers and conditions (20, 26). PCR products were purified and Sanger sequenced (Eurofins, Germany). The Ethics Committee of Shiraz University of Medical Sciences approved the study protocol and patients gave written consent to participate in the study (code: IR.SUMS.REC.1396.540).

## Results

The sequence of *NLRP7* and *KHDC3L* were analysed by Chromas software (Technelysium Pty Ltd, Australia). BLAST of sequences was performed for two genes based on the reference sequences in the NCBI database (*NLRP7*, NG_008056.1, and *KHDC3L*, NG_031942.1). Sequencing analysis of *NLRP7* in the patient revealed a new three nucleotides deletion in exon 4 in a homozygous state (Fig .1B). Sequence analysis of the patient’s sister with one spontaneous abortion and one normal child showed a heterozygous deletion status for these three nucleotides (Fig .1C). Normal sequence is provided in Figure 1D. This deletion is expected to remove amino acid Threonine in codon 185 (*c.555_557delCAC, p.Thr185del*) . The mutation was evaluated by parameters of Mutation Taster (www.mutationtaster.org) and it was regarded as disease-causing alteration. In addition, the mutation was analysed by PROVEAN parameter (http://provean.jcvi.org). Variants with a score equal to or below -2.5 are considered "deleterious," and variants with a score above -2.5 are considered "neutral." PROVEAN score was estimated -13.000 for this mutation. This means that the mutation is deleterious.

**Fig 2:**
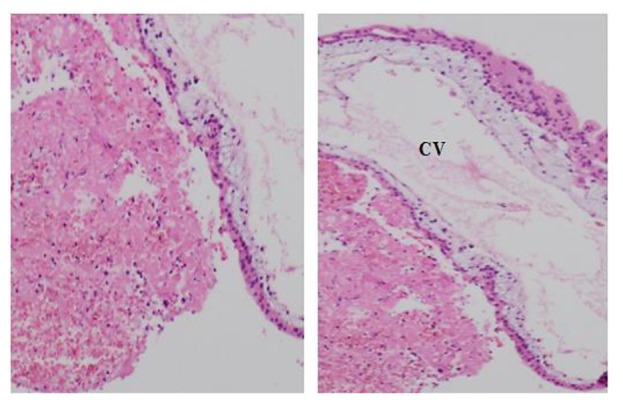
Photomicrograph of molar tissue from the patient. Excessive proliferation of trophoblastic tissue is seen around chorionic villi (CV) by hematoxilin/eosin staining histopathology analysis (magnification: left: × 100, right × 40).

In addition, Threonine in codon 185 is conserved in various species using multiple sequence alignment by Clustal Omegam (www.ebi.ac.uk/Tools/msa/clustalo/) (Fig .1E). Histopathology of the molar tissue for the patient is provided in Figure 2. Excessive proliferation of trophoblastic tissue has been observed around chorionic villi, while fetal tissues were clearly absent.

## Discussion

In this study, a new mutation in the homozygous state has been identified within the NACHT domain of NLRP7 protein, suggesting the importance of this domain in normal function. This study on a patient with a homozygous mutation in *NLRP7*, while she has a healthy boy via ovum donation, add further evidence that pathology of RHM is restricted to the oocyte and normal ovum is able to rescue defects of these patients for normal pregnancies. To date, four cases of ovum donation in patients with a mild missense mutations in *NLRP7* have been reported (27, 28). Investigations on healthy reproductive male individual with a homozygous mutation in *NLRP7* show that function of this gene is not necessary for normal sperm, in contrast to ovum (14, 15). The sister of indicated patient with heterozygous mutation has a spontaneous abortion and one normal child, confirming the impact of the defective allele of *NLRP7* on reproductive wastage, reported in recent finding (25).

## Conclusion

We report a new mutation in *NLRP7* gene, related to RHM and spontaneous abortion in homozygous and heterozygous states, respectively. Regarding this study and four previous reports, patients with homozygous mutation in *NLRP7* are able to have live birth with egg donation. In contrast to four previously reported cases with a mild missense mutations, investigation on this new patient shows that more deleterious mutations with severe functional effect are also good candidate for egg donation.
